# 79. Long-term Health Outcomes of Individuals who are Infected with SARS-CoV-2 and Develop COVID-19

**DOI:** 10.1093/ofid/ofac492.004

**Published:** 2022-12-15

**Authors:** Lilac Tene, Tobias Bergroth, Anna Eisenberg, Gabriel Chodick

**Affiliations:** Maccabitech, Maccabi Healthcare Services, Tel Aviv, Tel Aviv, Israel; MSD Sweden, Stockholm, Stockholms Lan, Sweden; MSD Israel, Hod Hashsaron, HaMerkaz, Israel; Maccabitech, Maccabi Healthcare Services, Tel Aviv, Tel Aviv, Israel

## Abstract

**Background:**

Persistence of symptoms after the acute phase of COVID-19, often referred to as long COVID, is common and debilitating. Data on the burden and direct medical costs of long COVID is still limited.

**Methods:**

A retrospective cohort study using data from a 2.6-million-member state-mandated health provider in Israel.

All adult patients with a positive SARS-CoV-2 RT-PCR test between March 2020 and March 2021 were included. Among them, patients with long COVID diagnoses or post-COVID symptoms persisting or newly diagnosed more than 4 weeks from the first positive RT-PCR test were defined as long COVID patients. Study endpoints included all COVID-related visits, hospitalizations, therapies, imaging, and lab tests. Costs of utilized healthcare service were evaluated for pre-infection, acute phase, and long COVID period.

**Results:**

Included in the study were 180,759 incident COVID-19 patients (32.9±19.0 years; 89,665 [49.6%] females). 14,088 (7.8%) individuals developed long COVID (40.0±19.0 years; 52.4% females). In addition to older age (adjusted odds ratio (AOR)=1.06, 95% CI 1.05–1.06), long COVID was associated with female sex (AOR=1.14, 95% CI 1.10–1.18), ever smoking (AOR=1.53, 95% CI 1.36–1.73), symptomatic acute phase (AOR=1.17; 95% CI 1.13–1.22); and hypertension (AOR=8.21, 95% CI 6.80–9.90), particularly among younger adults. Hospitalization in Long COVID patients was associated with respiratory/ear-nose-throat (adjusted hazard ratio (AHR)=2.55; 95% CI 2.27–2.86), neurological (AHR=2.88; 95% CI 2.33–3.56), psychiatric (AHR=2.32; 95% CI 1.93–2.79), and cardio-metabolic long-term complications (AHR=1.61; 95% CI 1.41–1. 83). While long COVID patients had a lower use of services compared to non-long COVID patients during the acute phase (AOR=0.87; 95% CI 0.83–0.90), their mean cost per user was 52% higher. Direct medical costs of long COVID patients were substantially greater than non-long COVID patients post vs. pre infection (AOR=1.74; 95% CI 1.56–1.93).

**Conclusion:**

This study demonstrated the association of long COVID with many complications requiring extended medical care and higher healthcare costs. These factors should be considered in priority setting around COVID-19 prevention and management.

**Disclosures:**

**Tobias Bergroth, PhD**, Gilead Sciences: Stocks/Bonds|MSD, Sweden: Employee|MSD, Sweden: Stocks/Bonds **Anna Eisenberg, PhD**, MSD, Israel: Employee|MSD, Israel: Stocks/Bonds.

